# Temporal lobe epilepsy and its negative impact on quality of life: could it improve through aerobic physical exercise?

**DOI:** 10.1055/s-0045-1814402

**Published:** 2026-03-12

**Authors:** Viviana B. Garcia Borja, Iraida C. Escalante Carranza

**Affiliations:** 1 Universidad Privada San Juan Bautista, Lima, Peru.

Dear Editor,

We write to you with great interest in the article titled “Aerobic physical exercise improves quality of life in temporal lobe epilepsy” by Laks et al. 1 The authors address an important topic related to the comprehensive management of patients with epilepsy, approaching the condition from a different perspective through the implementation of aerobic exercise.

The study describes a nonpharmacological treatment aimed at improving the quality of life (QoL) for these patients. As medical students in training, we value the exploration and promotion of physical exercise not only in epilepsy but also in various other conditions that affect public health today. Nonetheless, we would like to raise some concerns regarding the research.

Firstly, we note that while the study design is longitudinal, the specific period during which data collection occurred is not clearly stated. This omission limits our understanding of the temporal context in which the research was conducted. Additionally, the sample size appears to be insufficient to achieve adequate statistical power, as reflected in high *p* -values across most variables, making the findings less specific and conclusive. 2

Moreover, although randomized sampling is mentioned, the methodology used in this process is not clearly described. The study also lacks details on whether blinding methods or bias control strategies were employed, which could compromise the internal validity of the findings. Similarly, the inclusion and exclusion criteria are not fully defined, making it difficult to identify the studied population precisely and affecting the external validity of the results. Without clearly defined criteria, potential confounding factors may have influenced the outcomes. 3

We also found limited explanation regarding the tools used to assess QoL. Although internationally recognized instruments were applied, the rationale for their selection and their validity in detecting changes in patients with epilepsy were not adequately addressed. For conditions like epilepsy, which involve complex neurological mechanisms, studies should complement subjective questionnaires with objective evaluations, such as neurocognitive and neurophysiological markers, to better understand the disease's impact. 4

Lastly, the discussion section does not sufficiently explore the potential risks of engaging in physical exercise for individuals with epilepsy. While exercise may pose minimal risk for well-controlled patients, it could present complications for those in advanced stages of the condition. These concerns are often shared by patients, families, and healthcare professionals alike, and they should be addressed within educational strategies and future clinical research. 5

In conclusion, we acknowledge that this study represents a valuable step forward in exploring innovative nonpharmacological treatment approaches for epilepsy. However, from our perspective, higher methodological rigor is essential to ensure that such alternative therapies can be safely and effectively implemented in clinical practice, promoting patient-centered and evidence-based care.
